# Exit from totipotency in mammals: an epigenetic perspective

**DOI:** 10.3389/fcell.2026.1858789

**Published:** 2026-06-11

**Authors:** Sergio Ruiz

**Affiliations:** Laboratory of Genome Integrity, Center for Cancer Research/National Cancer Institute, National Institutes of Health (CCR/NCI/NIH), Bethesda, MD, United States

**Keywords:** ESC (embryonic stem cells), totipotency, totipotent-associated transcriptional program, transposable elements (TEs), ZGA

## Abstract

Totipotency is the ability of a single cell to generate a whole organism and is a property restricted to the cells of the earliest embryonic developmental stages. Interestingly, the process of zygotic genome activation (ZGA), which mediates in the embryo the switch from maternal inherited RNAs to embryonic transcripts, has been associated with the loss of totipotency potential. The exit from totipotency correlates with the quick and efficient silencing of a totipotent-associated stage-specific transcriptional program triggered during ZGA. Importantly, the timely and efficient silencing is required for proper embryonic development. However, the molecular mechanisms underlying the activation and subsequent silencing of this program remain elusive. This review will focus on an overlooked aspect associated with ZGA in mice that involves the control over the transient expression of the totipotent-associated transcriptional program. Furthermore, we will discuss the level of conservation of these mechanisms in humans.

## Introduction

In mammals, the fertilization of an oocyte by a sperm results in the formation of a totipotent zygote (Riveiro and Brickman, 2020). Totipotent cells hold the potential to develop a complete organism, including all embryonic and extra-embryonic lineages, and in mice, this is a property strictly restricted to zygotes and blastomeres of the 2-cell (2C) stage embryo (Schulz and Harrison, 2019; Jukam et al., 2017; Kojima et al., 2025). Further cleavages promote a progressive decrease in the developmental potential of the blastomere, which transitions to the pluripotent state observed in cells from the inner cell mass of the pre-implantation embryo (Schulz and Harrison, 2019; Jukam et al., 2017; Kojima et al., 2025). Interestingly, exit from totipotency correlates with the initiation of embryonic transcription, a process known as zygotic genome activation (ZGA). In mice, ZGA occurs in two waves of transcription, a minor wave at the end of the zygotic phase and a major phase during the mid-to-late 2C-stage embryo (Aoki, 2022). Because of the initiation of ZGA, a well-defined transcriptional program associated with totipotency (2C-associated program) is transiently expressed at the embryonic 2C-stage (Hendrickson et al., 2017; De Iaco et al., 2017; Whiddon et al., 2017). This program is characterized by the expression of stage-specific genes, such as members of the *Zscan4a-f* gene cluster, the *Pramef* family or the transcription factors (TFs) *Dux* and *Zfp352*, as well as transposable elements (TEs) of the murine endogenous retrovirus-L (MERVL) family (Hendrickson et al., 2017; De Iaco et al., 2017; Whiddon et al., 2017). The expression of these TEs is not a byproduct of ZGA, but it plays a crucial role in regulating the expression of hundreds of genes during this window of embryonic development (Sakashita et al., 2023). Importantly, while a defective activation of the 2C-associated program promotes embryonic arrest at the 2C stage, a failure in silencing its expression at the 4C stage results in a similar arrest (Guo et al., 2019). Hence, the relevance in efficiently silencing the totipotent program to proceed with further development. In humans, embryonic development is delayed compared to mice, with low levels of transcription that can be detected from the zygote to the 4C-stage embryo, and a major wave of transcription occurring at the 8C-stage (Schulz and Harrison, 2019; Jukam et al., 2017; Kojima et al., 2025). A similar transient totipotent-associated transcriptional program in human embryos is triggered during ZGA, including the expression of stage-specific TEs (Hendrickson et al., 2017; De Iaco et al., 2017; Whiddon et al., 2017).

In summary, this review will summarize the latest findings that explore the highly dynamic chromatin environment affecting the expression of the totipotent-associated transcriptional program during ZGA and its subsequent silencing.

## Transcriptional regulators of the totipotent program during ZGA in mice

**FIGURE 1:**
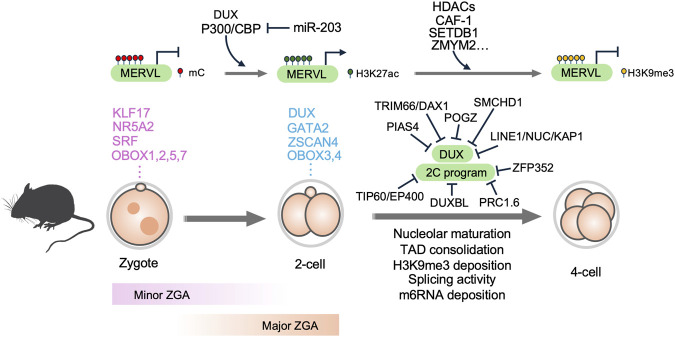
Schematic representation of the early embryonic development in mice summarizing the molecular pathways, specific proteins and epigenetic events involved in the silencing of the totipotent-associated transcriptional program following ZGA (note that the role of some of these has only been shown *in vitro*). MERVL elements undergo DNA demethylation following fertilization and, after a short activation at the 2C embryo stage characterized by H3K27ac deposition, they become permanently silenced upon H3K9 trimethylation. Maternal TFs (pink) and embryonic TFs (blue) are shown in the corresponding embryonic stage.

The transcriptional awakening of the genome is a highly orchestrated process that requires the timely translation of TFs from accumulated maternal RNAs. In mice, maternal KLF17, as well as members of the OBOX family (OBOX1/2/5/7), play a crucial role in RNA Pol II pre-configuration at the zygotic stage, facilitating the expression of major ZGA genes (Hu et al., 2024; Ji et al., 2023). Similarly, TFs and co-factors such as YAP, NFYA, NR5A2, SMARCAD2, CYCLIN T2, KDM1A, SRF or RFX1, among others, are essential for preparing the embryonic genome to initiate transcription during ZGA (Lu et al., 2016; Yu et al., 2016; Gassler et al., 2022; Zhang et al., 2022; Ancelin et al., 2016; Wang et al., 2022; Hermant et al., 2025; Figure 1).

At the end of the zygotic stage, the expression of TFs from the embryonic genome is then required to coordinate the major wave of transcriptional activity during ZGA (Kojima et al., 2025). TFs such as GATA2, zygotically expressed members of the OBOX family (OBOX3/4), ZSCAN4 or DUX are required to prime the embryo for further development by transiently activating the 2C transcriptional program (Choi et al., 2017; Ji et al., 2023; Hendrickson et al., 2017; De Iaco et al., 2017; Whiddon et al., 2017; Sakamoto et al., 2024; Figure 1). Although the loss of any of these factors individually is dispensable for ZGA, the combined loss, such as DUX and OBOX4 (Chen and Zhang, 2019; Guo et al., 2024), impairs development, suggesting functional redundancy among TFs. Most of our current knowledge on how the 2C program is activated and subsequently repressed is derived from studies on the TF DUX. Forced expression of DUX in mouse embryonic stem cells (mESCs) induces the generation of 2C-like cells (2CLCs), which show transcriptional and chromatin-associated characteristics of 2C embryonic blastomeres. Importantly, unperturbed cultures of mESCs contain a small percentage of 2CLCs, although it is unclear what mechanism underlies this endogenous conversion (Macfarlan et al., 2012). Although the full totipotent potential of the 2CLCs has not been strictly demonstrated by the generation of a full mouse from a single cell, 2CLCs are considered totipotent-like cells as they contribute to the trophectoderm (Macfarlan et al., 2012). The 2CLC model has been profusely used in the field to examine the molecular mechanisms regulating the induction, but also the dissolution, of the 2C program in an *in vitro* model (Genet and Torres-Padilla, 2020).

The extinction of the 2C program is a highly efficient and coordinated process (Fu et al., 2020a). The expression levels of DUX are tightly restricted to the early 2C stage by the repressive effect of the TRIM66/DAX1 complex, the SUMO E3 ligase PIAS4, the TF POGZ, the epigenetic regulator SMCHD1 as well as the physical recruitment of the *Dux* loci to perinucleolar heterochromatin upon nucleolus maturation (Yan et al., 2019; Ruebel et al., 2019; Dion et al., 2019; Yu et al., 2021; Zuo et al., 2022; Xie et al., 2022; Sun et al., 2023; Figure 1). Furthermore, LINE1 RNA has been shown to recruit the NUCLEOLIN/KAP1 complex to the *Dux* loci to impair its expression (Percharde et al., 2018). Indeed, LINE1 knockdown in zygotes induces a 2C embryonic arrest characterized by aberrant silencing of the DUX-induced transcriptional program (Percharde et al., 2018). In addition, DUX triggers a negative feedback loop by promoting the expression of the TF DUXBL. DUXBL seems to act as a dominant negative competing with DUX for the same binding sites at genes and MERVL elements (Vega-Sendino et al., 2024). It also recruits the TRIM24/33 repressor complex, which correlates with increased H3K9me3 deposition at DUX-induced transcriptional clusters Vega-Sendino et al., 2024). It is unclear whether DUXBL can interfere with the OBOX-induced transcriptional program, as OBOX TFs can also bind and activate MERVLs. Furthermore, the N6-methyladenosine (m6A) reader YTHDC1 has been shown to act upstream of SETDB1 to repress TEs and *Dux* (Liu et al., 2021). The transcription factor ZFP352, highly expressed at the 2C-stage and induced by DUX, has been shown to facilitate the dissolution of the totipotent state as its depletion delays the transition to morula (Li et al., 2023; Figure 1). Mechanistically, ZFP352 interacts with DUX and co-binds MT2-Mm TEs in the early 2C embryo, but further accumulation following initial induction switches its binding affinity to SINE/B1/ALU retroelements. These act as regulatory elements facilitating the expression of adjacent genes such as TRIM43, an E3-ubiquitin ligase protein, which was shown to ubiquitinate ZSCAN4 and DUX, facilitating exit from totipotency (Li et al., 2023). This work illustrated the relevance of different families of retroelements orchestrating a timely transition during early stages of development. ZSCAN4 has been shown to mediate the degradation of LSD1, HDAC1 and SUV39H1/2 by recruiting the ubiquitin ligase TRIM25, promoting chromatin opening and facilitating the 2C state (Yang et al., 2024). Finally, SUMOylation is relevant to suppress DUX expression in mESC through the recruitment of sumoylated PRC1.6 and KAP1/SETDB1 repressive complexes to *Dux* loci (Cossec et al., 2018; Figure 1). However, the role of SUMOylation *in vivo* remains to be determined.

In summary, different maternal and embryonic TFs participate in a timely manner to promote the activation of the totipotent transcriptional program and its subsequent dissolution.

## Epigenetic remodeling upon the exit from the totipotent state in the mouse embryo

During fertilization, the embryo requires extensive epigenetic remodeling in both parental genomes to create a permissive chromatin environment compatible with the rapid transcriptional changes occurring during ZGA and subsequent embryonic development. These changes involve global DNA demethylation, histone modifications, genome spatial reorganization and substantial transcriptional changes (Eckersley-Maslin et al., 2018; Fu et al., 2020b; Chen et al., 2023; Sotomayor-Lugo et al., 2024; Vega-Sendino and Ruiz, 2024; Kojima et al., 2025).

To establish totipotency and proper lineage specification, an extensive reprogramming of the DNA methylation landscape is required upon fertilization (Greenberg and Bourc’his, 2019). The parental genome undergoes rapid and active demethylation at the zygotic stage, driven by the methylcytosine dioxygenase TET3, while the maternal genome is passively demethylated through cell division (Shen et al., 2014). However, DNMT1/DNMT3A-dependent *de novo* DNA methylation in zygotes has also been observed (Amouroux et al., 2016). This genome-wide DNA demethylation reprogramming opens a window of opportunity for certain families of retroelements to be transiently expressed. However, these retrotransposons must be efficiently silenced to ensure proper development and histone modifications play an essential role in this silencing (Walter et al., 2016; Wang et al., 2018). MERVL and IAPEY3 TE switch from DNA methylation to H3K9me3-dependent repression following the exit from the embryonic 2C-stage (Wang et al., 2018). The H3K9 methylase SETDB1 seems responsible for this methylation as the maternal deficient embryo shows a failure to extinguish MERVL expression (Zeng et al., 2026). Furthermore, the chromatin assembly factor-1 (CAF-1) has been involved *in vitro* and *in vivo* in controlling the levels of H3K9me3 deposition and expression at LTR-derived families (Ishiuchi et al., 2015; Wang et al., 2018). In addition, CAF-1 participates in the canonical deposition of the histone H3.3 in the 2C embryo (Ishiuchi et al., 2021). This is important as H3.3 deposition at heterochromatin has been postulated to favor a chromatin permissive state (Ishiuchi et al., 2021). Interestingly, *Dux*-knockout embryos showed defective establishment of H3K9me3 at targeted LTRs either directly or through the regulation of zinc finger proteins (Xu et al., 2022). In addition, H3K27me3 also plays a role in the silencing of a set of LTR-derived families (Wang et al., 2018). Taken together, the epigenetic silencing of LTRs following ZGA is controlled by a switch from DNA methylation to histone methylation (Figure 1).

Zygotic chromatin is globally hyperacetylated, and it is characterized by broad H3K27ac domains that correlate with similarly broad H3K4me3 domains (Zhang et al., 2016; Wang et al., 2022). These H3K27ac domains prevent premature gene expression in zygotes, and HDAC activity is needed for the transition of broad H3K27ac to canonical peaks after the first cleavage. The activity of the histone acetyltransferases P300/CBP is required for H3K27ac deposition at the zygote and prime ZGA promoters and MERVL elements for activation (Wang et al., 2022). Exit from totipotency requires deacetylation at totipotent-associated genes and MERVLs, and the TRIM24/33 complex might recruit HDACs to these genomic regions (Vega-Sendino et al., 2024). Furthermore, the atypical polycomb repressor complex 1.6 (PRC1.6), which catalyzes the ubiquitination of histone H2A on Lysine 119 (H2AK119Ub) and possesses HDAC activity, as well as the chromatin remodeler TIP60/E400, have been shown to negatively regulate the 2C-transcriptional program *in vitro* (Rodriguez-Terrones et al., 2018).

Splicing activity has also been shown to regulate negatively the totipotent-transcriptional program. Indeed, chemical inhibition of splicing strongly induces the formation of 2CLC (Yang et al., 2022). Interestingly, 2C-stage embryos are characterized by defective splicing activity which seems restored after ZGA (Wyatt et al., 2022). MERVL RNAs are enriched in m6A, and although this modification has been shown to facilitate clearance of ERV-derived RNA species, the role of m6A on MERVLs *in vivo* seems unclear (Chelmicki et al., 2021; Wang et al., 2023).

MicroRNAs (miRs) also control the exit from totipotency. Loss of miR-203 induced embryonic developmental delay correlating with persistent expression of 2C-associated genes, including MERVL elements (González-Martínez et al., 2026). Interestingly, P300 was shown to be a direct target of miR-203 and critical to fine-tune the activity of P300 at MERVL elements which showed elevated levels of H3K27ac and H3K9ac upon miR-203 loss (González-Martínez et al., 2026). Furthermore, DUX-induced miR-344 targets the zinc finger protein ZMYM2 and LSD1, leading to MERVL transcriptional activation (Yang et al., 2020). ZMYM2 is critical to recruit the HDAC corepressor complex to mediate MERVL repression as ZMYM2 knockdown compromises the exit from totipotency (Yang et al., 2020).

During ZGA, the chromatin undergoes extensive three-dimensional organization and remodeling (Zhang and Xie, 2022; Ing-Simmons et al., 2022). Extensive weakening of compartments and topologically associated domains (TADs) characterizes the fertilized embryo (Zhang and Xie, 2022; Ing-Simmons et al., 2022). However, following ZGA, chromatin slowly refolds to consolidate TADs in a transcription-independent manner (Ke et al., 2017; Du et al., 2017). The lack of defined compartmentalization and TADs seems to be a general feature associated with totipotency (Olbrich and Ruiz, 2022). In fact, loss of SCC1, a key subunit of the cohesin complex, improves somatic cell nuclear transfer through partial de-repression of key minor ZGA genes (Zhang et al., 2020). Furthermore, depletion of the transcription factor CTCF in mESCs leads to efficient 2CLC conversion *in vitro* (Olbrich et al., 2021). The establishment of defined chromatin architecture and the formation of TADs can either facilitate promoter-enhancer interactions leading to robust gene expression or insulate certain genomic regions, promoting their silencing.

Overall, the exit from totipotency is a highly regulated process involving the coordinated effort of multiple layers to ensure a quick and efficient silencing of the 2C-transcriptional program (Figure 1).

## Exit from totipotency in the human embryo

**FIGURE 2:**
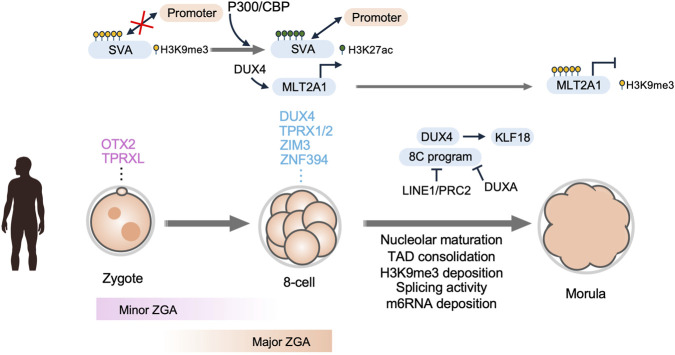
Schematic representation of the early embryonic development in humans summarizing the molecular pathways, specific proteins and epigenetic events involved in the silencing of the totipotent-associated transcriptional program following ZGA (note that the role of some of these has only been shown *in vitro*). SVA elements undergo the loss of H3K9me3 following fertilization and, after a short activation at the 8C embryo stage characterized by H3K27ac deposition, they become permanently silenced following H3K9me3 reinstallation. Maternal TFs (pink) and embryonic TFs (blue) are shown in the corresponding embryonic stage.

ZGA in humans is regulated by a combination of maternally deposited TFs, including the PAIRED (PRD)-like homeobox factors OTX2 and TPRXL (Zou et al., 2022; Wang et al., 2025; Figure 2), as well as embryonic TFs such as DUX4, ZIM3, ZNF394, TPRX1/2, LSM1, and ZNF675 (Zou et al., 2022; Nykänen and Vuoristo, 2024; Guo et al., 2025; Li et al., 2025; Firouzabadi et al., 2025; Figure 2). DUX4 induces totipotent-associated genes and a set of TEs, including HERVL and MLT2A1 repeats (Hendrickson et al., 2017). Recently, the TF KLF18 has been identified as a target downstream of DUX4 relevant for the expression of KLF17 and a subset of DUX4-induced genes (Hamm et al., 2026; Figure 2). In addition, DUX4 induces the expression of its paralog DUXA, which competes for the same target genes in a similar negative feedback loop as shown for mouse DUXBL (Bosnakovski et al., 2023; Figure 2).

Fertilization in human embryos also triggers an extensive wave of demethylation (Okae et al., 2014; Smith et al., 2014; Guo et al., 2014). However, single cell methylome sequencing showed thousands of genomic regions undergoing *de novo* methylation, demonstrating the existence of a dynamic balance (Zhu et al., 2018). Of note, strong DNA methylation occurs at the 8C-stage in regions enriched for ALU elements, suggesting that the transient activation of these elements during ZGA needs to be constrained to allow further development (Zhu et al., 2018). Human embryos are also characterized by a relaxed chromatin conformation, which gets strengthened following ZGA in a CTCF- and transcription-dependent manner (Chen et al., 2019). Human 8C-stage embryos are also characterized by defective splicing activity (Wyatt et al., 2022). Accordingly, chemical inhibition of splicing induces the formation of totipotent-like cells *in vitro* (Li et al., 2024).

Human embryos at the 2C and 4C-embryo stage are also characterized by genome-wide H3K27ac broad domains, which overlap with similarly broad H3K4me3 domains (Wan et al., 2026). These broad domains transition to narrow H3K27ac peaks by ZGA-dependent active deacetylation and decorate stage-specific transcriptionally active genes and transposable elements. Importantly, preventing deacetylation prior to ZGA impairs development and promotes embryo arrest (Wu et al., 2023). Indeed, reduced expression of the TFs divergent paired-related homeobox (*DPRX*) and arginine-fifty homeobox (*ARGFX*) leads to impaired ZGA by aberrant retention of histone acetylation (Guo et al., 2025). This suggest that the presence of broad H3K4me3/H3K27ac domains might prevent premature transcription in the zygote. Furthermore, newly deposited H3K27ac at genes and TEs during ZGA depends on the activity of P300, as its knockdown interferes with gene and transposon expression (Wan et al., 2026).

The stage-specific transcription of transposable elements is highly regulated during human embryo development. The hominoid-specific non-LTR SINE-VNTR-Alu (SVA) family of transposable elements undergoes H3K9me3 reprogramming and is decorated with H3K27ac during ZGA to facilitate the interaction with certain promoters to induce gene activation (Yu et al., 2022; Figure 2). Indeed, the SVA elements acting as enhancers are enriched in sequence motifs for the 8C TFs DUXA and ZSCAN4 (Yu et al., 2022). It is unclear how these elements are regulated following ZGA. In addition, LTR regions, but not non-LTR retroelements, undergo a similar regulation as their mouse counterparts, where DNA methylation is progressively substituted by H3K9m3 following ZGA (Xu et al., 2022). Importantly, H3K27me3 seems to play a minor role in LTR silencing during early human embryo development (Xu et al., 2022). Whether DUX4 or additional 8C TFs have a specific impact on H3K9me3 deposition, similar to what was observed in mouse embryos, is currently unknown. MLT2A1 elements of the HERVL LTR family, which are transiently activated by DUX4 during ZGA, generate chimeric RNA transcripts with other repeats and long non-coding RNAs with the ability of recruiting Pol II to specific ZGA-associated genes (Hendrickson et al., 2017; Xiang et al., 2026). These elements also undergo H3K9me3 deposition to promote their silencing upon exit from totipotency (Xu et al., 2022). m6A is also deposited in human retrotransposon RNAs although its effect on their stability and expression depends on the embryonic developmental time (Su et al., 2026).

Long interspersed nuclear elements-1 (LINEs) also play a crucial role in the exit from totipotency. By using 8C-like cells (8CLCs), an *in vitro* totipotent-like human model (Taubenschmid-Stowers and Reik, 2023), it was shown that RNA from members of the L1PA subfamily of LINE1 elements undergoes m6A in a METTL3-dependent manner. This methylation suppresses P300 binding through L1PA RNA to ERV1 TEs as well as to 8C enhancers and enhances KAP1 binding to ERVL-MaLR TEs, impairing their activity (Zhu et al., 2025). Furthermore, LINE1 RNA and PRC2 in 8CLCs contribute to maintaining H3K27me3 levels specifically in chromosome 19, which is enriched in 8C regulators and found to be associated with the nucleolus. This association prevents the expression of these regulators as LINE1 knockdown is associated with a relocation of chromosome 19 away from the nucleolus, allowing reactivation of the totipotent-associated program (Zhang et al., 2025; Figure 2). Importantly, the role of LINE1 elements suppressing the 8C program *in vivo* remains to be determined.

In summary, while significant progress has been made in the last few years to further understand human embryo development, much work is still needed. Nevertheless, the use of 8CLCs seems a good *in vitro* surrogate model to study the ongoing molecular events around ZGA.

## Discussion

The exit from totipotency in the mammalian embryo is a robust process that requires a series of epigenetic events perfectly timed to coordinate the transition towards pluripotency. DNA methylation, histone modification, chromatin compaction, post-transcriptional modifications, and 3D genome organization orchestrate a choreography of changes to convert a highly plastic and chromatin-permissive state to a more structured and restrictive chromatin configuration to limit developmental potential and set the stage for lineage segregation (Figures 1, 2).

In the coming years, the development of new technologies and the improvement of the existing ones will provide us with a better understanding of how embryos exit from totipotency in mammals.
